# Simulating human walking: a model-based reinforcement learning approach with musculoskeletal modeling

**DOI:** 10.3389/fnbot.2023.1244417

**Published:** 2023-10-12

**Authors:** Binbin Su, Elena M. Gutierrez-Farewik

**Affiliations:** ^1^KTH MoveAbility Lab, Department of Engineering Mechanics, KTH Royal Institute of Technology, Stockholm, Sweden; ^2^Department of Women's and Children's Health, Karolinska Institutet, Stockholm, Sweden

**Keywords:** human and humanoid motion analysis, motion synthesis, optimization, optimal control, kinematics, CMA-ES, reflex-based control

## Abstract

**Introduction:**

Recent advancements in reinforcement learning algorithms have accelerated the development of control models with high-dimensional inputs and outputs that can reproduce human movement. However, the produced motion tends to be less human-like if algorithms do not involve a biomechanical human model that accounts for skeletal and muscle-tendon properties and geometry. In this study, we have integrated a reinforcement learning algorithm and a musculoskeletal model including trunk, pelvis, and leg segments to develop control modes that drive the model to walk.

**Methods:**

We simulated human walking first without imposing target walking speed, in which the model was allowed to settle on a stable walking speed itself, which was 1.45 *m*/*s*. A range of other speeds were imposed for the simulation based on the previous self-developed walking speed. All simulations were generated by solving the Markov decision process problem with covariance matrix adaptation evolution strategy, without any reference motion data.

**Results:**

Simulated hip and knee kinematics agreed well with those in experimental observations, but ankle kinematics were less well-predicted.

**Discussion:**

We finally demonstrated that our reinforcement learning framework also has the potential to model and predict pathological gait that can result from muscle weakness.

## 1. Introduction

In recent years, reinforcement learning (RL) (Sutton and Barto, 2018) has emerged as a promising approach for motion synthesis such as human walking where an agent learns to adapt its behavior through interacting with the environment. Many optimization techniques used to develop controllers for simulated locomotion are based on reinforcement learning. RL algorithms have been applied in several studies to develop torque-driven control for physically simulated articulated models. Peng et al. (2018) used RL to generate a set of human movements including walking and running. Schulman et al. (2015) simulated dynamic gaits using high-dimensional, general-purpose neural network function approximators for both the policy and the value function in a variety of robot models. Duan et al. (2016) presented a benchmark suite of continuous control tasks with a simple humanoid based on a systematic evaluation of their effectiveness in training deep neural network policies. Even though RL algorithms can successfully develop controllers capable of performing a versatile set of locomotion tasks, the resulting behaviors generally appear less natural than normal human movements (Heess et al., 2017; Rajeswaran et al., 2017; Peng et al., 2018). More specifically, controllers trained with RL have exhibited large upper body motion, abnormal gaits, and unrealistic body posture (Heess et al., 2017). One of the reasons stems from the absence of biomechanical models that take into account the excitation and contraction of the muscles, the geometry and inertia properties of the body segments, and the external forces from the environment. Musculoskeletal models (Zajac, 1989; Thelen et al., 2003) represent a sophisticated dynamical system comprised of bones as articulating rigid bodies and muscles as actuators. These models often account for the neural excitation of muscles and also muscle contraction dynamics, which are determined by muscles' optimal lengths, shortening/lengthening velocities, and activations. Muscle contraction generates muscle force which is then transmitted to the bone through a compliant tendon. Muscle force causes joint torques to the body segments, thus generating human motions.

Musculoskeletal models can mainly be driven by three locomotion control frameworks: trajectory tracking (Neilson and Neilson, 1999; Fey et al., 2012), optimal control (Pandy et al., 1995; Suzuki, 2010) and reflex-based control (Geyer and Herr, 2010). In trajectory tracking, the controller solves the optimization problem by reducing the squared error between simulated and predefined trajectories and the squared muscle activation over a specific time interval, outputting the required muscle activation to mimic the predefined movement (Silverman and Neptune, 2012). Computed muscle control is a popular approach to estimate muscle activation that generates motion that in turn tracks the desired trajectory (e.g., joint angles from experimental motion capture) (Thelen et al., 2003). However, the method merely reproduces the predefined trajectory and cannot predict responses to new inputs. In optimal control, the controller solves the optimization problem by minimizing a specific cost function (e.g., metabolic energy expenditure or summed muscle activations) while achieving a task-objective function such as a steady-state gait. This control method is free from experimental data but requires sufficient domain knowledge to craft a cost function and represent natural human movement, which can also make it more computationally expensive (Anderson and Pandy, 2001). Performance criteria in predictive simulations with complex musculoskeletal models are frequently based on energy (Minetti et al., 1994) or muscle activity (Miller et al., 2012) minimization, but which criterion best represents reality remains unclear, and its formulation may vary depending on the musculoskeletal model. Recent review articles in predictive simulations of human movement describe both the potential and the challenges involved in realistic application in pathological motion (De Groote and Falisse, 2021). Novel applications of optimal control are emerging to predict optimal orthosis properties for persons with gait pathology (Febrer Nafría et al., 2022). In reflex-based control, the controller determines muscle activations and hypothesized reflex pathways to generate joint torques that drive the musculoskeletal model, mimicking human gait while optimizing a cost function (e.g., minimal metabolic cost or maximal walking distance). The muscle excitation is associated with computed muscle length or muscle force feedback while the reflex pathways accommodate leg mechanics to prevent joint hyperextension and maintain gait stability (Seyfarth et al., 2001; Günther et al., 2004). The reflex-based model does not require input from a predefined movement and can perform a natural walking motion by interacting the muscles and reflex pathways with the physics-based environment. Geyer and Herr (2010) presented a 2D human model controlled by reflex that can perform stable walking through interaction with the ground, while it tolerates ground disturbances and adapts to slopes without parameter interventions. Their approach can also predict some individual muscle activation patterns from experimental data. They further extended the model to a 3D locomotion study and compared neural controls for 3D-related motions by adding degrees of freedom at the hips in the frontal plane (Song and Geyer, 2013). Song and Geyer (2015) further developed this model by incorporating a higher layer, longer latency control that can alter some of the reflex gains. The added layer can adjust the desired foot placements and identify which leg to switch into swing control during double support. In a similar manner, Eilenberg et al. (2010) used an adaptive muscle-reflex controller for powered ankle-foot prostheses to adapt to environmental disturbances such as speed transients and terrain variation. Clinical trials have been successfully conducted with a transtibial amputee walking on level ground, ramp ascent, and ramp descent conditions. Thatte et al. (2018) implemented a reflex-based control policy on five subjects walking with a powered knee and ankle prosthesis and found that the level-ground walking torque and angle profiles from the prosthesis are similar to those of a weight and height-matched subject with intact limbs. Sharbafi et al. (2018) developed a control algorithm of an exoskeleton with one biarticular actuator based on a reflex-based human walking model that employs leg force to adjust hip compliance.

This study aims to use model-based RL methods to develop control modes that can produce realistic human walking in a musculoskeletal model driven by 18 muscle-tendon units. The main novel contribution of this study is the RL-based approach to solve the control parameters in a complex musculoskeletal model, as well as the design of the reward functions that can generate stable walking gaits reasonably similar to those of able-bodied persons in terms of joint kinematics and muscle activation patterns at different walking speeds or even with muscle weakness. While inspired by the work of Song and Geyer (2015), we apply a different reward function in that we introduced a pelvis component to encourage the model to walk naturally with a reasonable pelvis tilt angle. We formulated the human walking problem as a standard Markov decision process (MDP). We modeled the policy with a reflex-based controller to output muscle excitation that eventually activates the muscles. The MDP problem was solved and the controlled parameters were optimized with derivative-free covariance matrix adaptation evolution strategy (CMA-ES). We then generated gait without imposing target walking speed, i.e., allowing the model to settle on a stable walking speed. We also generated gait with a range of imposed target walking speeds. All simulations were performed without reference data from motion capture. We finally demonstrated the model's potential to predict pathological gaits, in this case, gait that may result from muscle weakness.

## 2. Methods

We used an integrated OpenSim-RL (Kidziński et al., 2018) platform which embedded OpenSim (Delp et al., 2007) and OpenAI Gym (Brockman et al., 2016) to simulate muscle-driven forward movement in a physics-based simulation environment. Experimental data were collected and used solely for comparison with simulation outcomes.

### 2.1. Reinforcement learning

**Figure 1:**
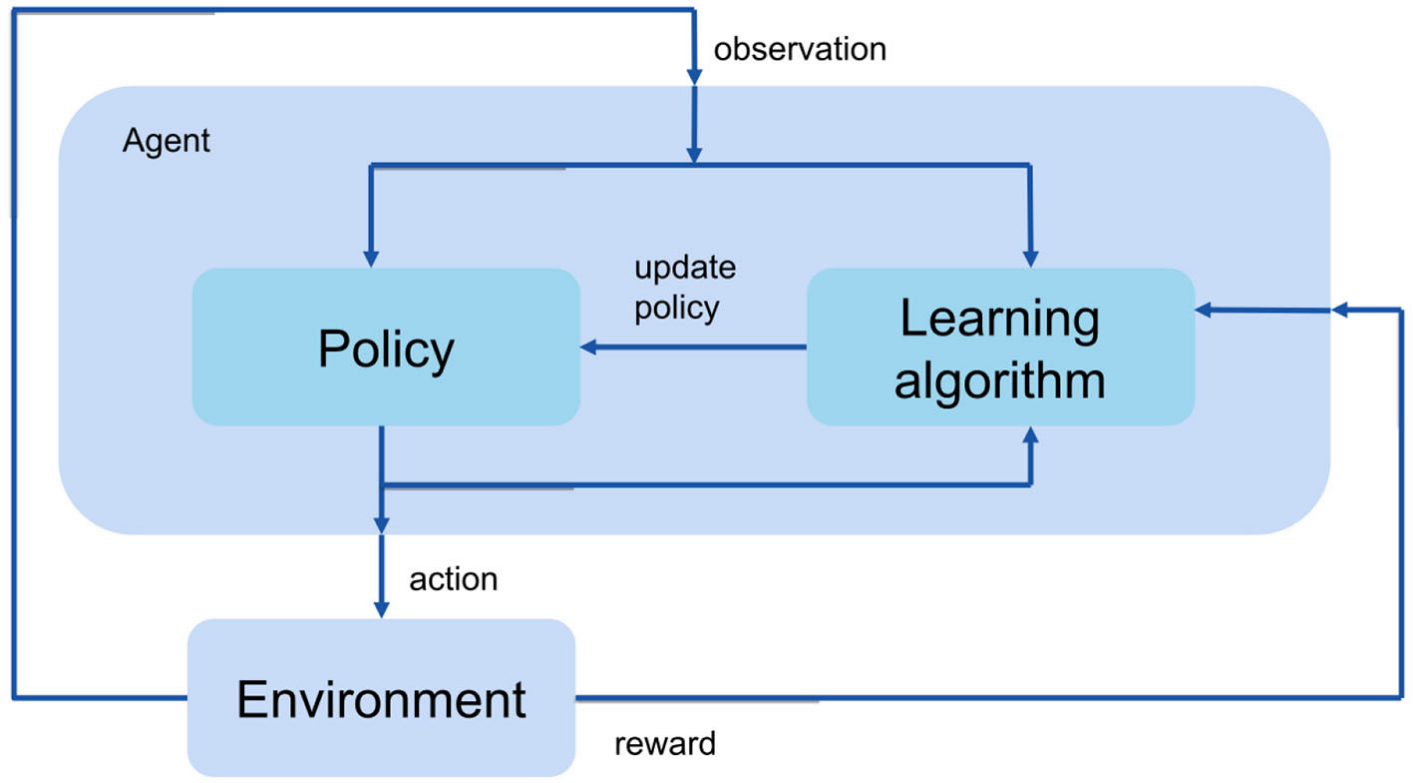
An illustration of the RL flow.

The goal of reinforcement learning is to train an agent to complete a task. The agent receives observations and a reward from the environment and sends actions back to the environment. In the current study, the environment is a musculoskeletal model that has 9 joint degrees of freedom and 18 muscles (Figure 1). The observations contain movement information such as joint position, velocity, ground contact, etc. The actions are the muscle excitations of each muscle. The agent contains two components: a policy and a learning algorithm. The policy produces actions based on the observations from the environment. The learning algorithm continuously updates the policy parameters based on the actions, observations, and reward.

### 2.2. Musculoskeletal model

**Figure 2:**
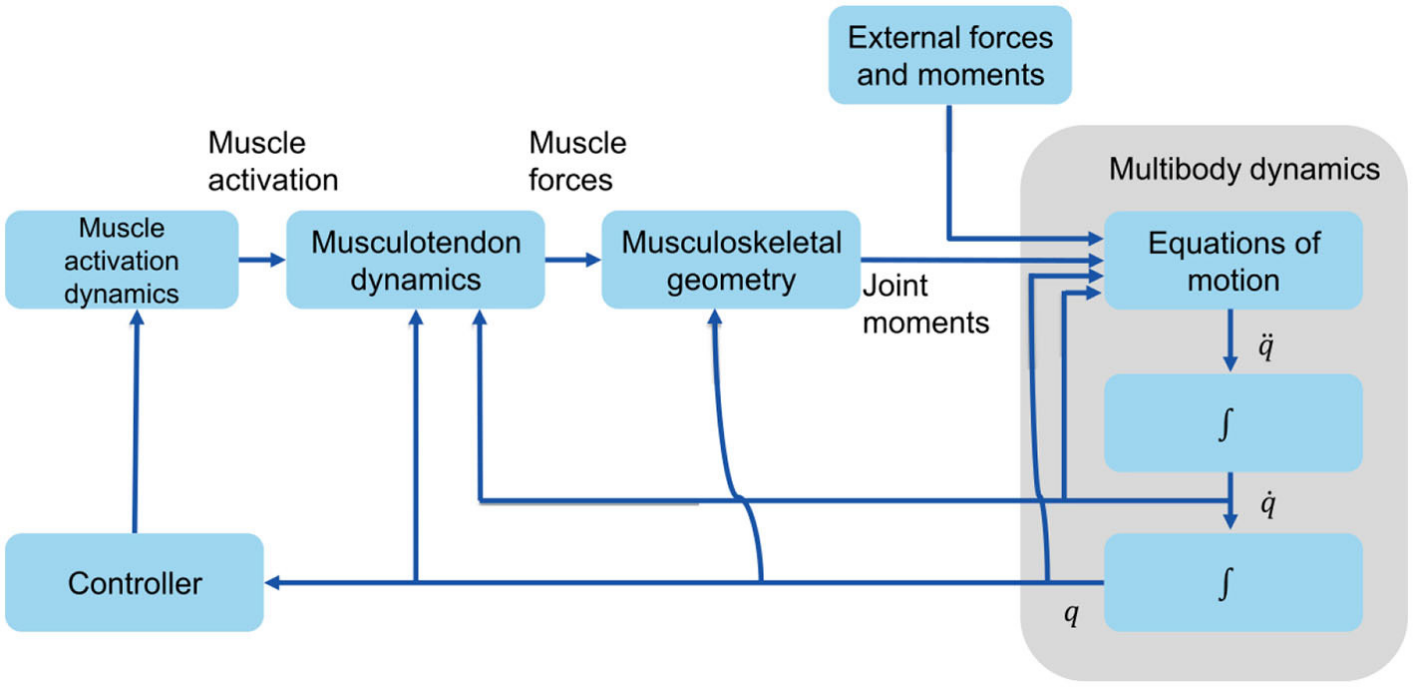
Forward dynamics that depict the production of human movement. *q*, $\dot{q}$, and $\ddot{q}$ are vectors of the generalized coordinates, velocities, and accelerations, respectively.

The musculoskeletal model in this paper is a simplified 2D model adapted from Delp et al. (1990), with 6 internal degrees of freedom: flexion/extension at the hips, the knees, and the ankles, and 18 Hill-type muscle-tendon units: iliopsoas (ILPSO), gluteus maximus (GMAX), hamstrings (HAM), rectus femoris (RF), vasti (VAS), biceps femoris short head (BFSH), gastrocnemius (GAS), soleus (SOL), and tibialis anterior (TA) for each leg. Muscle parameters and moment arms are according to Delp et al. (1990), and tendons were assumed as non-compliant. OpenSim's forward-dynamics approach was used, in which the musculoskeletal system has muscle excitations as inputs and outputs the body motions (*q*, $\dot{q}$, and $\ddot{q}$) (Figure 2). Since muscle cannot activate or relax instantaneously, there is a delay between muscle excitation, muscle activation, and the development of muscle force. This delay is modeled by the model activation dynamics (Zajac, 1989). The musculotendon dynamics (Anderson and Pandy, 1993) describe the translation of muscle activation to muscle force. The musculoskeletal geometry determines the muscles' moment arms. Joint moments are determined from muscle forces and moment arms. Finally, through multibody dynamics (Kane and Levinson, 1983), accelerations, velocities, and angles for each joint are computed.

### 2.3. Markov decision processes (MDP) formulation

We formulated the forward-dynamics simulation of the musculoskeletal model as an MDP <S,A,R> which consists of the set of body states S, possible muscle excitation A, and the expected rewards $R_{s,s^{\prime }}^{a}$ received when going from state *s* to *s*′ ($s,s^{\prime }\in S$) after performing action *a*. The body states S are joint position, velocity, ground contact, etc. We considered that the agent accumulates rewards through interacting with the environment. The agent follows its actions according to a deterministic policy π:S→A which indicates how action *a* is chosen in state *s*. Our goal was to find an optimal policy π such that the expected future reward is maximized. In our case, the policy was modeled by a gait controller based on a reflex-based framework (Song and Geyer, 2015) for human locomotion that maps the body states to muscle excitation.

### 2.4. Reward design

A forward-dynamics simulation was run by integrating the musculoskeletal model's dynamic equations starting from a user-specified initial state. Muscle states were set by equilibrating the force between the muscle and tendon at an activation based on the excitations calculated by the gait controller. Then, new states at a small time interval (0.01*s*) were determined by numerical integration until the desired simulation time was reached or the pelvis of the human model fell below 0.6 m. During simulation, the agent gathered survival rewards (*R*_*alive*_) and footstep rewards (*R*_*steps*_). The total reward is high when the human model locomotes at desired velocities with minimum muscle effort and pelvis tilt.

(1) $$\begin{array}{l} \begin{array}{l} r=R_{alive}+R_{steps}\\ \;=\overset{t}{\underset{i}{\sum }}r_{alive}+\\ \;\overset{steps}{\underset{j}{\sum }}\left(w_{steps}r_{steps}-w_{vel}J_{vel}-w_{pel}J_{pel}-w_{mul}J_{mul}\right) \end{array} \end{array}$$

where *t* is the total simulation timestep, *r*_*alive*_ is the survival reward for each timestep *i* [i.e., *r*_*alive*_ is the sum of the timesteps (0.01s) if the simulation does not fail], *w*_*steps*_, *w*_*vel*_, *w*_*pel*_, *w*_*mul*_ are the weights for the step reward and velocity, pelvis tilt, and muscle effort costs. The values of these weights were determined by trial and error tests and finally set to 10, 60, 20, and 1, respectively. The survival reward *R*_*alive*_ encourages the model to search for solutions to stay alive throughout the simulation. The footstep reward *R*_*steps*_ evaluates gait behaviors within footsteps rather than at discrete instances of time, for example, to allow the model's walking speed to vary within a footstep, similar to how humans walk. Specifically, *r*_*steps*_ was designed to encourage the model to take footsteps but not unnecessarily small steps. *J*_*vel*_ penalizes movements that deviate from target speed. *J*_*pel*_ penalizes large pelvis tilt during locomotion. *J*_*mul*_ minimizes muscle excitations and distributes the load to muscles more efficiently. Thus, the rewards and costs within footsteps are defined as:

(2) $$\begin{array}{l} \begin{array}{l} r_{steps}=\overset{t_{step}}{\underset{ii}{\sum }}\Delta t\\ \;J_{vel}=|\overset{t_{step}}{\underset{ii}{\sum }}\left(v_{pel}-v_{tgt}\right)\Delta t|\\ \;J_{pel}=\overset{t_{step}}{\underset{ii}{\sum }}\theta_{pel}^{2}\Delta t\\ \;J_{mul}=\overset{t_{step}}{\underset{ii}{\sum }}\overset{muscles}{\underset{k}{\sum }}e_{k}^{2}\Delta t \end{array} \end{array}$$

where *t*_*step*_ is the number of simulation timesteps in one footstep, *ii* is the *ii*th timestep in one footstep, Δ*t* is the simulation interval 0.01*s*, *v*_*pel*_ and *v*_*tgt*_ are the velocity of the pelvis and the target velocity respectively, θ_*pel*_ is the pelvis tilt, *e*_*k*_ is the muscle excitation of the *k*th muscle.

### 2.5. Covariance matrix adaptation evolution strategy (CMA-ES)

We used CMA-ES (Hansen et al., 2003), which represents the population by a full-covariance multivariate Gaussian, to solve the MDP problem including 37 control parameters for the gait controller and 12 parameters for the model's initial states. The control parameters are the target angles of the trunk, knee, and ankle, force feedback, length feedback, velocity feedback, proportional-derivative feedback and co-stimulation of the muscles. These 12 initial states are the forward speed, rightward speed, pelvis height, trunk lean angle, hip abduction/adduction, hip flexion/extension, knee flexion/extension, and ankle dorsiflexion/plantarflexion for both sides (Song and Geyer, 2015). We set the population size to be 16 for each generation and ran for 1,000 generations for every trial. The parameters were updated every 16*th* simulations whenever a higher reward was encountered. In every generation, the CMA-ES will generate 16 simulations with different values of control parameters in parallel. Based on the highest reward achieved in these simulations, the model will then seed the control parameters for the next 16 simulations. The generation number was set large enough that the MDP problem could usually be solved at the end of each trial. To accelerate the optimization process, we established a common E2 virtual machine with 8 vCPUs and 32 GB memory on the Google cloud platform to run parallel optimizations with the same initial parameters. The best solution from the previous generation was used to seed the next generation of optimizations (Algorithm 1).

**Algorithm 1:**
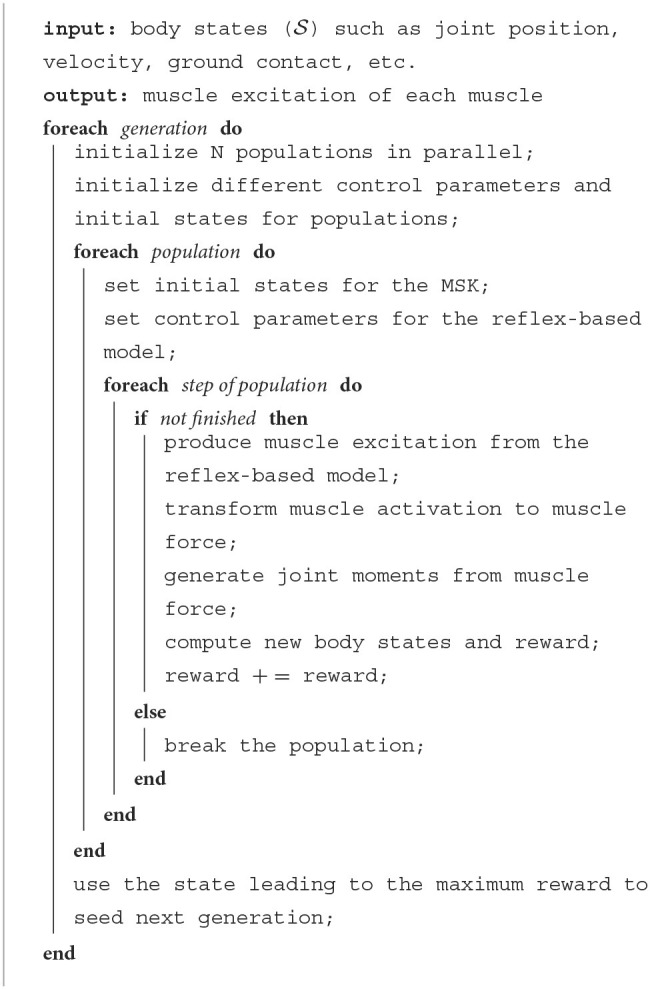
Training algorithm structure and description.

### 2.6. Evaluation

We evaluated the performance of our simulation in 3 aspects: generating gaits without a prescribed gait speed, simulating gaits in a range of speeds, and simulating gait impairment with reduced maximum isometric force (MIF) of SOL and GAS at a speed of 1.45 *m*/*s*. Experimental data from 8 able-bodied adults walking at several different speeds were used for visual comparison with the simulation results (Appendix). To evaluate the agreement of the simulated walking pattern at its natural speed 1.45 *m*/*s* to the observed gait kinematics at a mean speed of 1.41 *m*/*s*, correlation coefficients (R) between simulated and observed gait were computed for hip, knee, and ankle kinematics.

To generate gaits without a prescribed gait speed, we removed the velocity cost *J*_*vel*_ from the reward function and set the control parameters and initial states to random values. The simulation time for each simulation was set to be 20 *s*. We then found the solution with CMA-ES with parallel computing. The steady walking speed developed during the simulation was 1.45 *m*/*s*. Films illustrating the incremental learning process can be viewed in the Supplementary material.

To generate simulations of gait at a range of target speeds from 1.1 to 1.8 *m*/*s*, we first solved the MDP problem in the prediction horizon of 10*s* using CMA-ES with parallel computing in GCP at a target speed of 1.45 *m*/*s*. In the initial optimization, the control parameters and initial states were randomly assigned, which means we assumed no prior knowledge of the problem, and the initial position of the model was not in consideration. After the first MDP was solved, the optimized parameters were used to seed the next optimization of the neighbor speeds until the lowest or highest speeds, e.g., from 1.45 to 1.27 *m*/*s*, then from 1.27 to 1.10 *m*/*s*.

To generate gait impairment with simulated muscle weakness of SOL and GAS, we used the solution previously solved at 1.45 *m*/*s* as the initial parameters for the controller. The MIF of SOL and GAS were set to 80 or 60% of the original value wherein only one muscle strength was reduced per simulation. The problem was solved via 4 simulations of impaired gait.

## 3. Results

We present the salient results of gait kinematic and kinetics, specifically the sagittal plane hip, knee, and ankle angles, the vertical ground reaction force (vertical GRF) and the muscle excitations for all evaluations. The gait cycle, including descriptions of foot rockers, is described here according to Perry and Davids (1992). Only the simulations with the highest reward were presented. All simulated data are presented only for the model's right side and normalized to one gait cycle except for the vertical GRF normalized to the stance phase. The number of simulated gait cycles varied between 10 and 15 depending on the walking speed, and results are shown as the ensemble average of these 10–15 gait cycles ± one standard deviation. Each optimization problem took between 12 and 18 h to complete.

### 3.1. Simulated gait without prescribed speed

**Figure 3:**
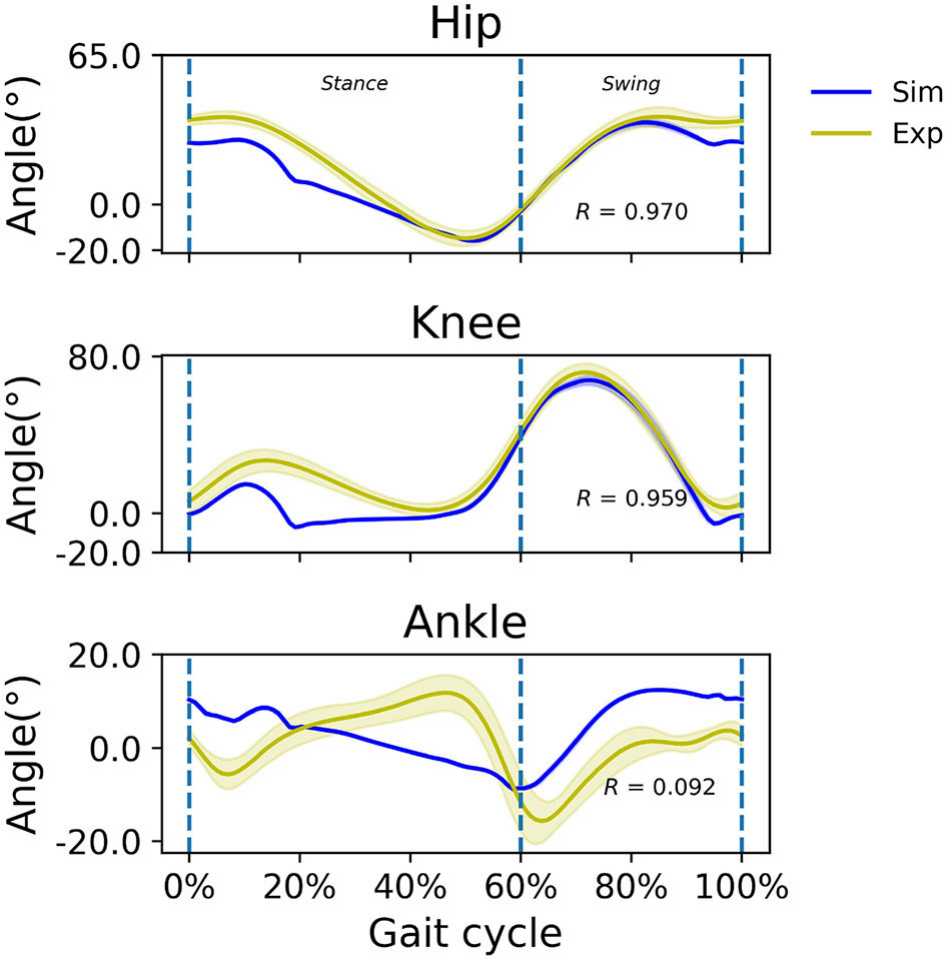
Simulated (blue) and experimental (yellow) sagittal plane kinematics of the hip, knee, and ankle at the simulated walking speed of 1.45 *m*/*s* and an average experimental walking speed of 1.41 *m*/*s*, respectively. Positive joint angles indicate flexion/dorsiflexion. Correlation coefficient *R* is indicated.

Without a prescribed speed, the model settled on a walking speed of 1.45 *m*/*s*; the simulated hip, knee, and ankle joint angles are illustrated (Figure 3). Experimental kinematics from able-bodied subjects walking at a comfortable speed, which was on average 1.41 *m*/*s*, is also illustrated. Hip and knee kinematics matched reported experimental observations reasonably well with correlation coefficients *R* = 0.97 and *R* = 0.96, respectively, with a somewhat better agreement in swing than in stance, but ankle kinematics were different from observed kinematics (*R* = 0.09). In the simulated ankle kinematics, the gait cycle began with a heel rocker (plantarflexion with heel contact during approximately 0–8% of the gait cycle), but the ankle rocker (dorsiflexion via tibial advancement over the ankle with whole-foot contact during approximately 8–30% gait cycle), forefoot rocker (dorsiflexion with forefoot contact during approximately 30–50% gait cycle) and toe rocker (rapid plantarflexion with forefoot/toe contact during approximately 50–60% of the gait cycle) were absent in the simulation. Instead, the ankle was dorsiflexed at foot contact, and continued to plantarflex more or less constantly until toe-off.

### 3.2. Simulated gait over a range of prescribed speeds

**Figure 4:**
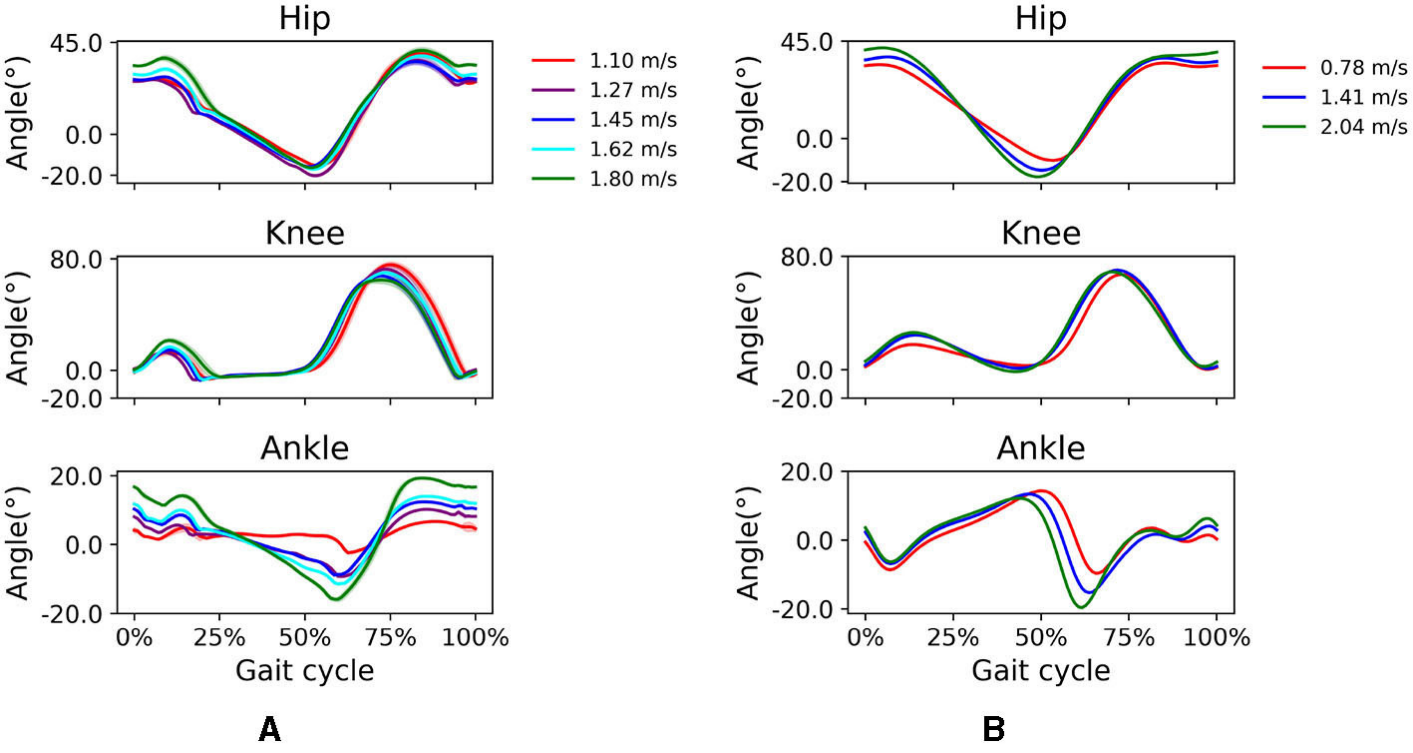
**(A)** Simulated and **(B)** experimental sagittal plane kinematics of the hip, knee, and ankle at different walking speeds. For the simulated data, walking speeds range from 1.1 to 1.8 *m*/*s*. For the experimental data, average walking speeds range from 0.78 to 2.04 *m*/*s*.

All simulations produced stable gait patterns at the five prescribed walking velocities between 1.1 and 1.8 *m*/*s* in the prediction horizon of 10 *s*. Simulated knee kinematics were more realistic at higher walking speeds than at lower speeds. The stance phase became relatively shorter as walking speed increased (toe-off shifted from 70 to 56% of the gait cycle). This temporal shift was more noticeable in the simulation. The joint kinematics showed expected trends as speed increased; peak flexion and extension in the hip and ankle increased with higher walking speeds as well as peak knee flexion in loading response (Figure 4). The timing of peak plantarflexion in pre-swing and peak knee flexion in swing was shifted temporally, reflecting the earlier toe-off. These kinematic trends agree reasonably well with observed trends in experimental data for subjects walking at 0.78 to 2.04 *m*/*s*.

**Figure 5:**
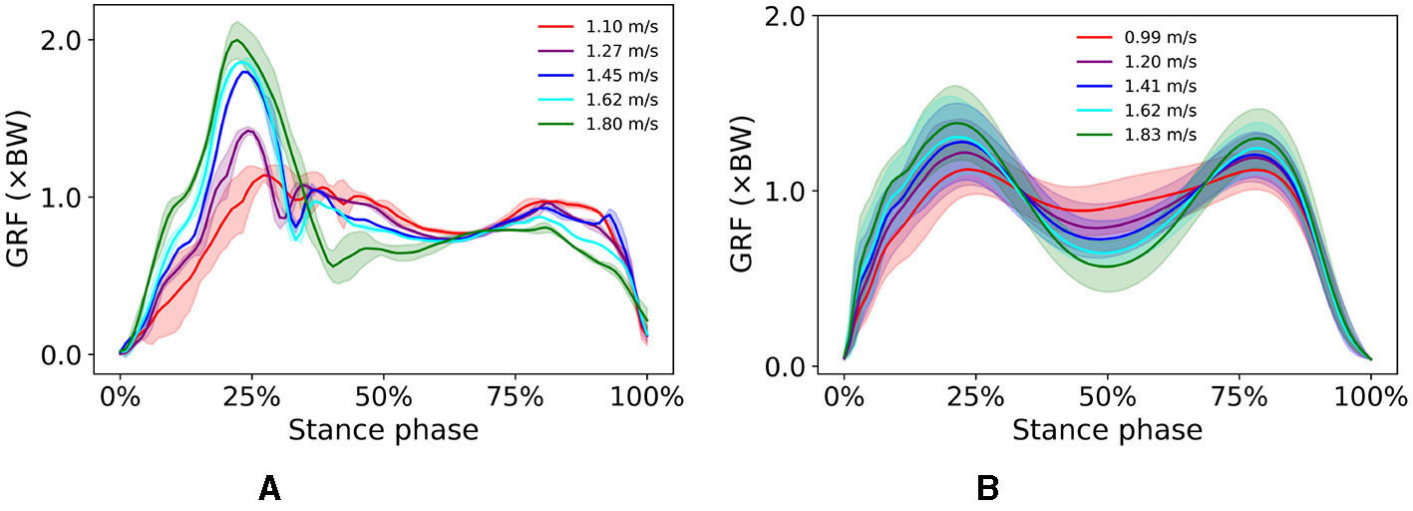
Vertical GRF of simulated walking **(A)** at different speeds ranging from 1.1 to 1.8 *m*/*s* and experimental walking **(B)** at different speeds ranging from 0.99 to 1.83 *m*/*s*.

Several features of the simulated vertical GRF are common to the experimental GRFs; in both, the first vertical peak increases and the minimum vertical GRF at mid-stance decreases with increasing walking speed (Figure 5). However, the expected second vertical GRF peak during pre-swing was not as prominent in simulated walking as in experimental data, and while it should increase with increasing speed, the simulated second vertical GRF peak actually decreased with increasing speed. This behavior has been observed by Keller et al. (1996) who indicated that walking at a higher speed can result in a lower second vertical GRF peak than at lower speeds.

**Figure 6:**
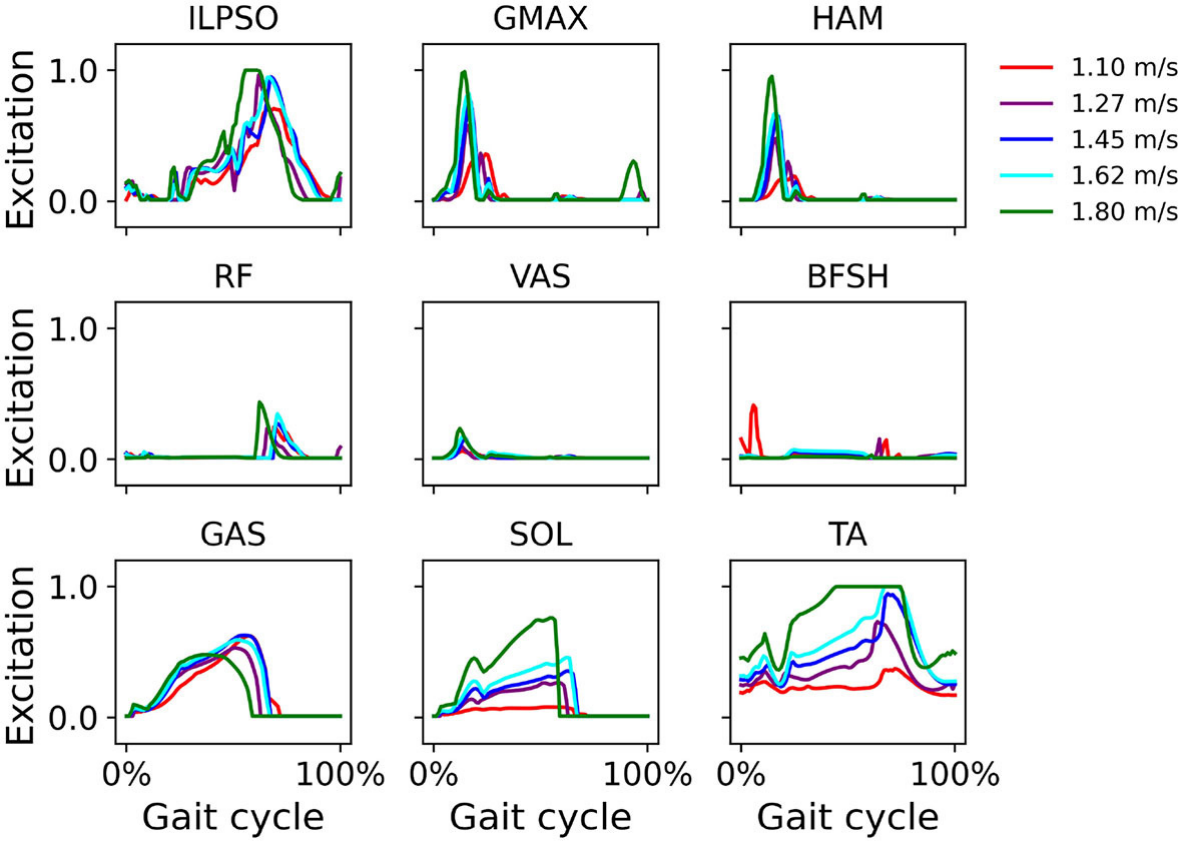
Muscle excitation during simulated walking at different speeds ranging from 1.1 *m*/*s* to 1.8 *m*/*s* in the iliopsoas (ILPSO), gluteus maximus (GMAX), hamstrings (HAM), rectus femoris (RF), vasti (VAS), biceps femoris short head (BFSH), gastrocnemius (GAS), soleus (SOL), and tibialis anterior (TA).

Computed muscle excitations indicate GMAX, HAM, and VAS activation during early stance and SOL and GAS activation during mid- and terminal stance (Figure 6). Excitation of major muscles such as ILPSO, GMAX, HAM, SOL, and TA increased with increasing walking speed.

### 3.3. Simulated gait with muscle weakness at 1.45 *m*/*s*

**Figure 7:**
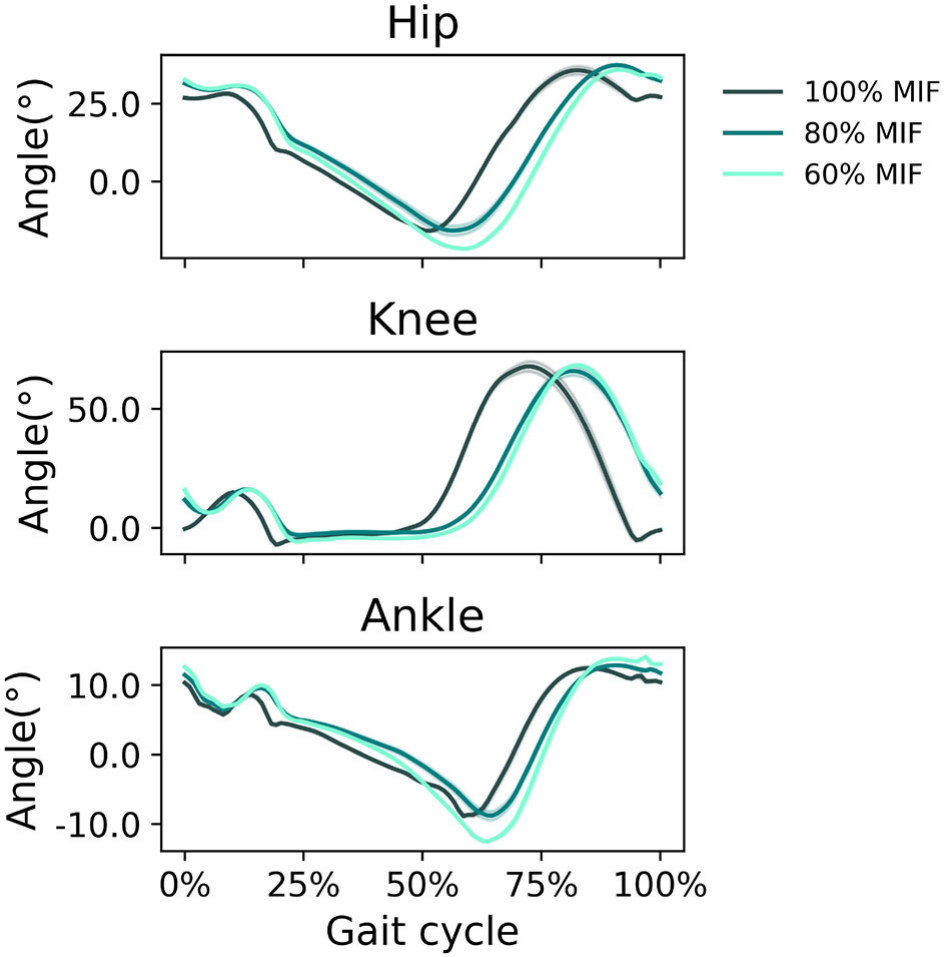
Sagittal plane angles of the hip, knee, and ankle during simulated walking at a speed of 1.45 *m*/*s* in 3 conditions: normal GAS strength, GAS strength decreased to 80% MIF, and GAS strength decreased to 60% MIF.

**Figure 8:**
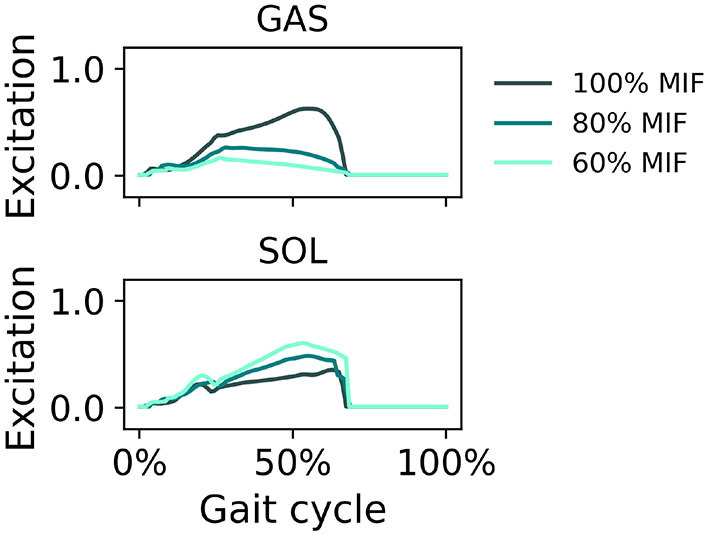
Muscle excitation of GAS and SOL during simulated walking at a speed of 1.45 *m*/*s* in 3 conditions: normal GAS strength, GAS strength decreased to 80% MIF, and GAS strength decreased to 60% MIF.

When GAS muscle weakness was simulated, the hip tended to extend more and the ankle tended to plantarflex more in pre-swing (Figure 7). With decreasing GAS strength, the SOL excitation increased and the GAS excitation decreased (Figure 8).

**Figure 9:**
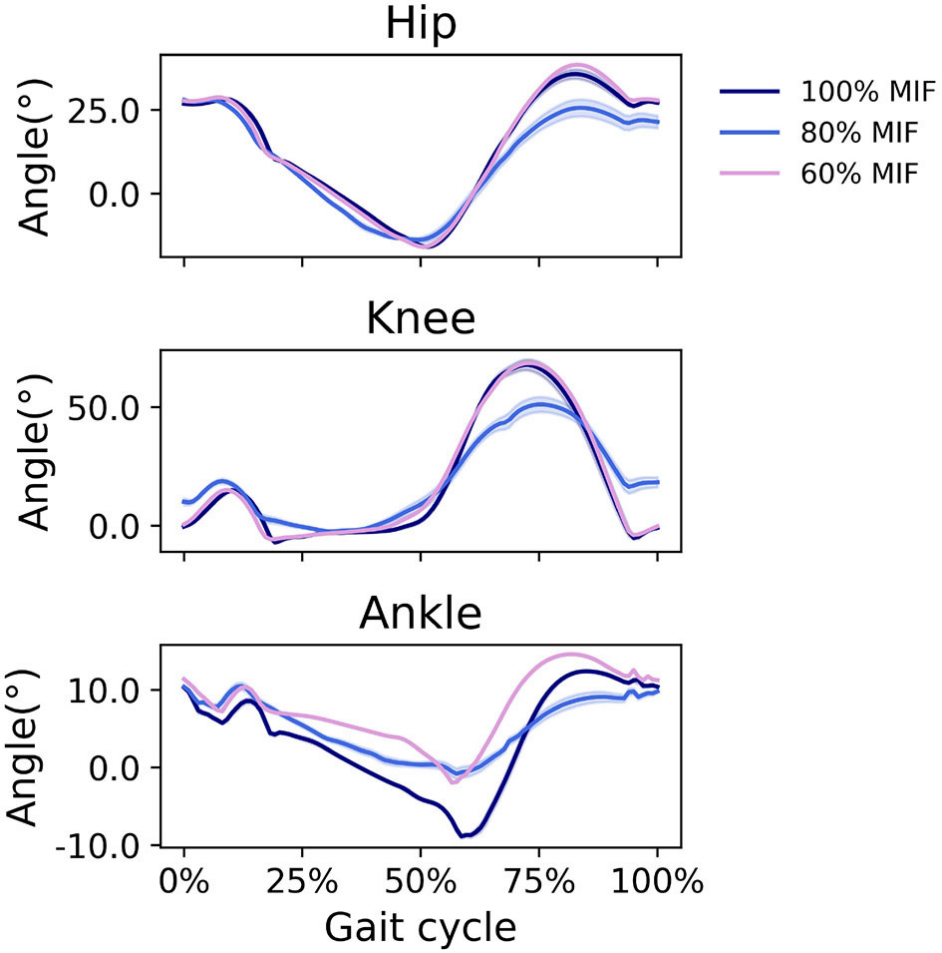
Sagittal plane angles of the hip, knee, and ankle during simulated walking at a speed of 1.45 *m*/*s* in 3 conditions: normal SOL strength, SOL strength decreased to 80% MIF, and SOL strength decreased to 60% MIF.

**Figure 10:**
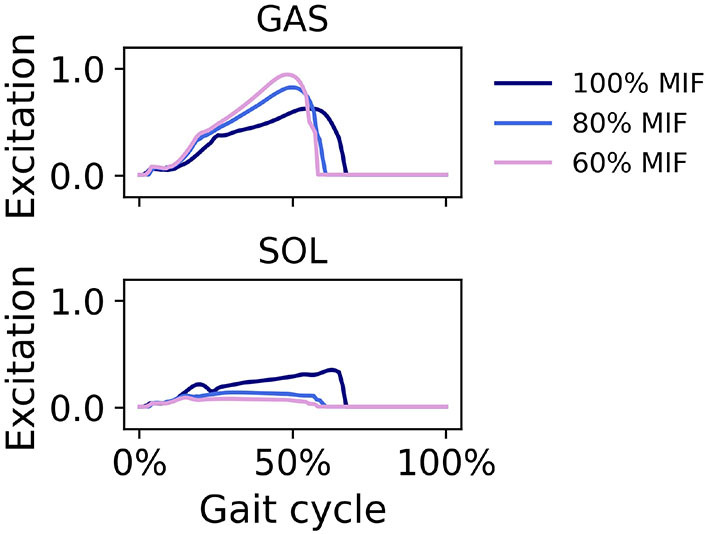
Muscle excitation of GAS and SOL during simulated walking at a speed of 1.45 *m*/*s* in 3 conditions; normal SOL strength, SOL strength decreased to 80% MIF, and SOL strength decreased to 60% MIF.

When SOL muscle weakness was simulated, the hip tended to flex more during swing, and the ankle plantarflexed less; the ankle did not reach a plantarflexed position when SOL strength was reduced to 60% MIF (Figure 9). The kinematic pattern shifted temporally to the left. With decreasing SOL strength, the SOL excitation decreased and the GAS excitation increased (Figure 10).

## 4. Discussion

In this study, we show that human walking can be reproduced with a musculoskeletal model realistically by solving the MDP problem using CMA-ES. The successful reproduction of human walking can be attributed to the reflex-based controller that mimics simple feedback laws based on sensory data accessible at the spinal cord, such as muscle length, speed, force, and foot contact information and the specialized reward function that can guide the model to achieve physiologically realistic waking. We experimented with different reward components, and found that adding pelvic tilt makes the model converge to the target speed faster and produce better joint kinematics. Pelvic tilt plays a critical role in determining the alignment of the spine, hips, and lower limbs, which in turn affects muscle activations, joint forces, and stability. Incorporating it into simulations enhances the realism of the model by accurately representing how the pelvis's orientation impacts the rest of the body's kinematics and kinetics. Particularly, the pelvis tilt component in the reward function can guide the model to maintain an upright upper body position, and increase muscle activation to achieve higher walking speed as required. Without this component, the model would conveniently tilt the upper body and use gravity to accelerate the speed, ultimately leading to either a fall or a less natural walking position. The controller adopted in our model outputs muscle excitation signals that correspond reasonably well with reported muscle activation during normal gait (Perry and Davids, 1992). These excitation signals could eventually generate biologically plausible torque patterns, whereas other controllers often employ inefficient or even impossible torque patterns for humans (Wang et al., 2012). However, not all muscle activities from our simulation (Figure 6) agree well with observed muscle activity during human gait, and do not corroborate previous literature using similar models (e.g., Geyer and Herr, 2010; Song and Geyer, 2015). This disagreement can at least in part be attributed to the complexity of rigid differential equations, combined with the sensitivity of movement simulations to changes in muscle excitations and kinematics, which makes predicting and optimizing movement patterns challenging. This is a common issue in fields that merge biomechanics, robotics, and physics simulations where accurate representation of dynamic systems is crucial. To overcome this issue, Falisse et al. (2019) used direct collocation to solve optimal control to reduce the sensitivity of the cost function. However, their simulated kinematics did not entirely agree with those observed experimentally. They suggest co-contraction can play a stabilizing role during walking, which does agree with our simulation. The computed gait kinematics, vertical GRF, and muscle excitations were compared and evaluated under a variety of target walking speeds. We even computed solutions with simulated muscle weakness of the plantarflexors SOL and GAS, to demonstrate the potential use of the simulations in clinical applications.

In the simulation without a prescribed gait speed, the model converged to a steady walking speed of 1.45 *m*/*s*, even with various random control parameters and initial states. This indicates that the model is robust enough to not be influenced by prior guesses of the system. Our result corroborates findings by Umberger et al. (2003) whose model eventually developed a constant walking speed, provided that the objective functions, e.g., a minimal error between simulated and reference trajectory and minimal muscle effort, were optimized. Ackermann and Van den Bogert (2010) and Miller et al. (2012) used a “predictive” approach without tracking experimental data, in which the data-tracking solution had to serve as an initial guess for the control variables. A major benefit of our approach is that the forward simulation is free from any reference data such as captured motion and GRF data, even for setting initial control variables. As for other RL approaches used to produce human-like walking with a musculoskeletal model, Song et al. (2021) report a simulated gait pattern that did not resemble a natural gait pattern. Similar to Song et al., the simulated ankle kinematics from our study did not agree well with observed ankle kinematics during experiments. For example, our simulation did not display ankle, forefoot or toe rockers. Our simulated gait did exhibit some expected phases, such as heel rocker and dorsiflexion during swing to achieve foot clearance (Figure 3). Our simulation also exhibited knee flexion during loading response, though somewhat less than observed data. We speculate that the model's knee joint reaction force is much higher than in reality. Since the reward function in our method does not penalize the joint reaction force, this can cause unnatural kinematics if a large joint reaction force is present.

In simulations over a range of walking speeds, our model was capable of developing stable gaits at different speeds with the prescribed walking speed in the reward function. We were able to predict expected temporal shifts toward the shorter stance phase with increasing walking speeds, as well as expected increases in hip and ankle sagittal plane motion and reduced gait variability at higher speeds (Figure 4). Our findings of gait variability agree with experimental findings by Terrier and Schutz (2003) who reported low intra-subject gait variability at preferred and high speeds, but higher variability at low walking speed. Schwartz et al. (2008) reported in an experimental study increases in maximum knee flexion during swing with higher walking speeds, which we did not see in our simulations. We attribute this to the small excitation of the knee flexor BFSH in the model. Ong et al. (2019) found a similar trend in their musculoskeletal simulations. The increased ankle plantarflexion at fast walking speeds suggests the control framework responded accordingly to find a solution to adjust the gait kinematics to a fast walking pace. The vertical GRF shows a local vertical GRF peak in loading response at all speeds and a greater standard deviation at 1.1 *m*/*s* than at higher speeds (Figure 5), which suggests that the control framework could more easily converge to steady gait solutions in medium and fast walking speeds than at slow speeds. In the muscle excitation predicted in our simulations (Figure 6), the hip extensors GMAX and HAM were activated in loading response, i.e., when the hip extended to advance the trunk over the support limb. The hip flexor ILPSO was activated during pre-swing and initial swing, resisting hip extension during stance and reversing the hip into flexion during swing. The knee extensors VAS and RF activated eccentrically to restrain knee flexion in loading response. The ankle plantarflexors GAS and SOL were activated during mid-stance, late stance, and preswing, i.e., when their activity is expected to first control tibial advancement then to propel the leg into swing. The dorsiflexor TA was active throughout swing, to contribute to foot clearance. The expected TA activity in loading response to prevent foot drop was, however, not predicted in our simulation. The expected hamstrings activation in late stance to decelerate the knee extension was also not predicted in our simulation.

In simulations in which muscle weakness in GAS and SOL was modeled, the produced gait kinematics were slightly different that with full muscle strength, wherein simulated SOL weakness influenced all kinematics, particularly ankle kinematics, more than GAS weakness (Figures 7, 9). This is likely attributable to the muscle parameters in the musculoskeletal model; the uniarticular SOL is stronger than the biarticular GAS in the model, i.e., the modeled SOL MIF is higher than the modeled GAS MIF. The model could more easily compensate for GAS weakness with minimal kinematic changes than for SOL weakness. According to van der Krogt et al. (2012) who simulated how muscle weakness can be compensated by synergies in normal walking, GAS weakness led to increased SOL activation, and SOL weakness likewise led to increased GAS activation. Compensations for the weakness of individual muscles included increases in activation in unimpaired muscles, but not necessary increases in the impaired muscle's activation, corroborating our findings in muscle activation in Figures 8, 10. While Ong et al. (2019) found decreased walking speed when weakness in plantarflexor muscles was simulated, attributed to reduced push-off force in pre-swing, our simulation indicates that the prescribed walking speed can still be maintained, as long as the synergistic ankle plantarflexor can compensate for the weak muscle. Unlike the study by Falisse et al. (2019) in which gait was simulated with muscle weakness by imposing gait symmetry over a complete gait cycle and reserve actuators to prevent the simulation from falling, our model did not assume periodicity of the gait cycle and included no reserve actuator or residuals to guarantee that the model could still achieve steady walking. Nevertheless, the model still managed to perform locomotive behaviors without non-physical compensatory forces commonly seen in other physics-based environments. It is worth pointing out that our simulations with weakness were created to demonstrate how this model can be applied to study optimal phenomena in walking with or without muscle weakness; while accurate and individualized representation of pathological gait is on the horizon, it will require individualized muscle parameters, accurate reproduction of internal and external forces, and possibly subjective factors that affect how a person interacts with the external environment.

There are some limitations in the current study. Our simulation was not able to accurately represent realistic ankle kinematics; we speculate that more realistic kinematics may be achieved through computing and incorporating joint reaction forces in the cost function and with a more sophisticated contact model. The musculoskeletal model used in the simulation was also limited in that it is a 2D planar model and can thereby not represent the full characteristics of gait. In the current study, the gait controller was based on a reflex-based framework (Song and Geyer, 2015), though modified to not activate hip abductors and adductors; we restricted motion to the sagittal plane only, as the reinforcement learning algorithm could not converge to identify optimal control parameters in 3D. This warrants future implementation using a 3D musculoskeletal model that at least accounts for more degrees of freedom such as hip ab/adduction and hip rotation, which can stabilize the hip in the frontal plane and allow foot clearance with a less sagittal plane hip range of motion. We only present limited simulations of walking in the present study, whereas simulation of different conditions such as inclined or uneven surfaces, which require further adjustments of the OpenSim-RL environment, can further challenge the robustness of the RL approach. Computational efficiency is another limitation and was not prioritized in this study; the aim of our approach was instead to build a bridge between musculoskeletal modeling and reinforcement learning. Direct collocation could tremendously reduce the computational cost, but it normally optimizes for one footstep and its implementation in this simulation to encode “robustness” in the solution may be challenging, whereas the single-shooting with CMA-ES in our study optimized for multiple steps as it naturally does.

## 5. Conclusion

We present a model-based RL approach to simulate realistic human walking in a musculoskeletal model, first allowing the model to settle on a stable speed, then given faster and slower target speeds. The computed kinematics, ground reaction forces, and muscle excitation patterns and trends correspond reasonably well with those from reported normal gait, as indicated by good correlation of hip and knee kinematics and by similar trends over a range of walking speeds, with exception to ankle kinematics, which were not realistic in simulations. We further generated pathological gaits that result from ankle plantarflexor muscle weakness using the same approach. Our simulation results illustrate that the proposed approach can reliably find solutions to perform steady locomotion that are not sensitive to the initial guess of the control parameters and states. The simulations were achieved in the absence of reference motion data from motion capture. With the proposed RL framework, neuromechanical simulations can be developed to model versatile human movements and predict human motor behavior.

## Data availability statement

The raw data supporting the conclusions of this article will be made available by the authors, without undue reservation.

## Ethics statement

The studies involving human participants were reviewed and approved by the Swedish Ethical Review Authority. The participants provided their written informed consent to participate in this study.

## Author contributions

BS formulated the problem, performed the simulation, analyzed the results, and drafted the original manuscript. EG-F supervised the research process and gave concrete advice during implementation. All authors critically reviewed the manuscript. All authors contributed to the article and approved the submitted version.

## Appendix

Eight able-bodied adults (5 men/3 women, mean ± standard deviation age: 37.8 ± 9.6 years old, height: 1.76 ± 0.10 m, body mass: 76.6 ± 14.4 kg) participated in this experiment. The experiments were approved by the Swedish Ethical Review Authority (Dnr. 2020-02311), and all participants provided written consent. Participation was voluntary and could be terminated at any time during the experiment. For each subject, cadence was recorded while subjects walked on a treadmill at 70, 85, 100, 115, and 130% of their preferred walking speed (PWS) in randomized order. The PWS was determined by the participant's gender, age, and height (Bohannon, 1997). Then, subjects walked along a 10-m pathway in an instrumented motion lab at five speeds by matching their cadences from the treadmill at each speed. Marker positions (100 Hz) and ground reaction force (1,000 Hz) were measured using optical motion capture (Vicon V16) and strain gauge force platforms (AMTI, Watertown, MA, USA), respectively. Full-body marker placement was implemented based on the Conventional Gait Model with the extended-foot model (CGM 2.4). Joint kinematics were calculated based on marker coordinates using the Inverse Kinematics (IK) Tool in Opensim (Delp et al., 2007). Between 5 and 10 gait cycles per person and speed were analyzed, and the side, i.e., left or right, was chosen at random for each person.
